# Longitudinal MR angiographic evaluation of circle of Willis morphologic remodeling and induced aneurysms in Hashimoto rat cerebral aneurysm model

**DOI:** 10.1038/s41598-026-37369-2

**Published:** 2026-02-03

**Authors:** Yeon Soo Kim, Sungbin Hwang, Mi Hyeon Kim, Boseong Kwon, Yunsun Song, Kyubong Lee, Deok Hee Lee

**Affiliations:** 1 Biomedical Engineering Research Center, Asan Institute for Life Sciences, Asan Medical Center, Seoul, Korea; 2 Department of Radiology, Asan Medical Center, Research Institute of Radiology, University of Ulsan College of Medicine, 88 Olympic-ro 43-gil, Songpa-gu, Seoul, 05505 Republic of Korea; 3 Department of Radiology, Asan Medical Center, University of Ulsan College of Medicine, Seoul, Korea

**Keywords:** Cerebral aneurysm, Circle of Willis, MR angiography, Vascular remodeling, Hemodynamic stress, Hashimoto rat model, Diseases, Medical research, Neurology, Neuroscience

## Abstract

We aimed to longitudinally characterize morphological remodeling of the Circle of Willis (COW) and aneurysm development in the Hashimoto rat cerebral aneurysm model using serial high-resolution 7T MR imaging. Aneurysm induction was achieved through unilateral carotid and renal artery ligation combined with salt and β-aminopropionitrile loading to induce hemodynamic stress and vascular fragility. TOF-MRA, T2-weighted, and black-blood MR sequences were acquired at four time points over 12 weeks. Morphological changes were quantified by measuring arterial diameters and tortuosity indices. Postmortem scanning electron microscopy (SEM) following vascular corrosion casting was used as the diagnostic reference. Significant asymmetric vascular remodeling was observed predominantly in the posterior circulation, with fusiform aneurysms confirmed by SEM in the left PCA P1 segment in two cases, both associated with subarachnoid hemorrhage. Microaneurysms below MR detection threshold were found at ACA–OA and ICA–MCA bifurcations. The sensitivity and specificity of MR-based aneurysm detection were 40% and 60%, respectively. While spatial resolution limited visualization of smaller lesions, longitudinal MR imaging allowed dynamic tracking of vascular changes and provided insight into early aneurysmogenesis. This platform enables in vivo evaluation of aneurysm initiation, growth, and rupture risk, supporting its potential utility for preclinical assessment of therapeutic strategies.

**Supplementary Information:**

The online version contains supplementary material available at 10.1038/s41598-026-37369-2.

## Introduction

Intracranial aneurysms are an important cerebrovascular pathology with an estimated prevalence of 2–5% in the general population^1^. While often asymptomatic, rupture of an intracranial aneurysm can lead to subarachnoid hemorrhage (SAH), a devastating form of stroke associated with high morbidity and mortality^2^. The unpredictability of aneurysm rupture and the limited therapeutic window after SAH highlight the need for deeper understanding of the mechanisms underlying aneurysm formation, growth, and rupture. Such knowledge is essential to improve patient stratification, optimize timing for prophylactic treatment, and identify novel therapeutic targets^3^.

To investigate the pathogenesis of cerebral aneurysms, a variety of small animal models have been developed. Among these, the Hashimoto rat model has been widely used for over four decades^4^. This model reproduces key pathological features of human aneurysms by inducing hemodynamic stress and vessel wall fragility through a combination of carotid and renal artery ligation, high-salt diet, and lysyl oxidase inhibition. While robust and reproducible, most studies employing the Hashimoto model rely on terminal endpoints such as vascular corrosion casting, histopathology, or cross-sectional imaging. Consequently, dynamic changes in cerebrovascular morphology—including arterial remodeling, aneurysm evolution, and rupture events—have largely been inferred from pooled group-level data, rather than directly visualized in vivo over time^5^.

Recent advances in high-resolution preclinical magnetic resonance imaging (MRI) technology have opened new possibilities for non-invasive assessment of cerebrovascular architecture in small animal models^6–8^. In particular, time-of-flight MR angiography (TOF-MRA) and black-blood imaging (BBI) now allow researchers to visualize cerebral vessels serially in vivo, with sufficient spatial resolution to track subtle morphological changes allowing multiple longitudinal examinations. These developments enable a transition from single-time-point to truly longitudinal studies in aneurysm research, thereby offering a new platform for studying the dynamics of arterial remodeling, aneurysm formation, and rupture within the same animal. Such in vivo approaches not only reduce inter-animal variability but also allow real-time correlation with physiological parameters such as blood pressure.

Leveraging recent advances in preclinical MR imaging, we hypothesized that longitudinal, repeatable high-resolution imaging would enable dynamic visualization of arterial morphological changes in the Circle of Willis (COW), including diameter alterations and tortuosity, as well as the induction, progression, and rupture of aneurysms, in a rat model subjected to unilateral carotid ligation under hypertensive conditions.

The aims of this study were threefold: (1) to characterize time-dependent vascular remodeling in the COW using serial in vivo MR angiography, (2) to evaluate the diagnostic accuracy of MR imaging for aneurysm detection using scanning electron microscopy (SEM) as the reference standard, and (3) to analyze the incidence, morphology, and timing of aneurysm-related events (AREs) including rupture in Hashimoto rat cerebral aneurysm model.

## Results

### Surgical success rate and final analytical cohort

Surgical success was defined as the successful completion of the planned vascular manipulations and the animal’s survival until the acquisition of high-quality MR imaging at the first follow-up time point (W1). Two rats in the induction group were classified as surgical failures. One animal died immediately after surgery due to a surgical-related cause, while the other developed a massive infarction following the procedure and was found dead prior to the first follow-up scan.

Based on this definition, 13 out of 15 rats (*n* = 15) met the criteria, yielding a surgical success rate of 86.7%. These 13 rats in the induction group were included in the final analysis.

### Longitudinal imaging completion and reasons for early dropout

Successive MR imaging was performed at four time points: before surgery (W0, *n* = 13, excluding the 2 rats with surgical failure), post-surgery week 1 (W1, *n* = 13), week 4 (W4, *n* = 13), and week 12 (W12, *n* = 8). All 13 rats that underwent successful surgery completed imaging through W4. However, imaging at W12 was not obtained for 5 rats due to early mortality. Among them, 3 rats died from SAH, while the remaining 2 died of unknown causes. Despite comprehensive postmortem evaluations, including imaging and cranial autopsy, no definitive intracranial abnormalities were identified in these two cases. Ultimately, 8 rats survived until W12 and completed both final MR imaging and postmortem assessments, including SEM or histological evaluation. In contrast, all 6 rats in the control group survived through W12 and successfully underwent MR imaging at every scheduled time point.

### Longitudinal variability of blood pressure on induced hypertension

**Fig. 1:**
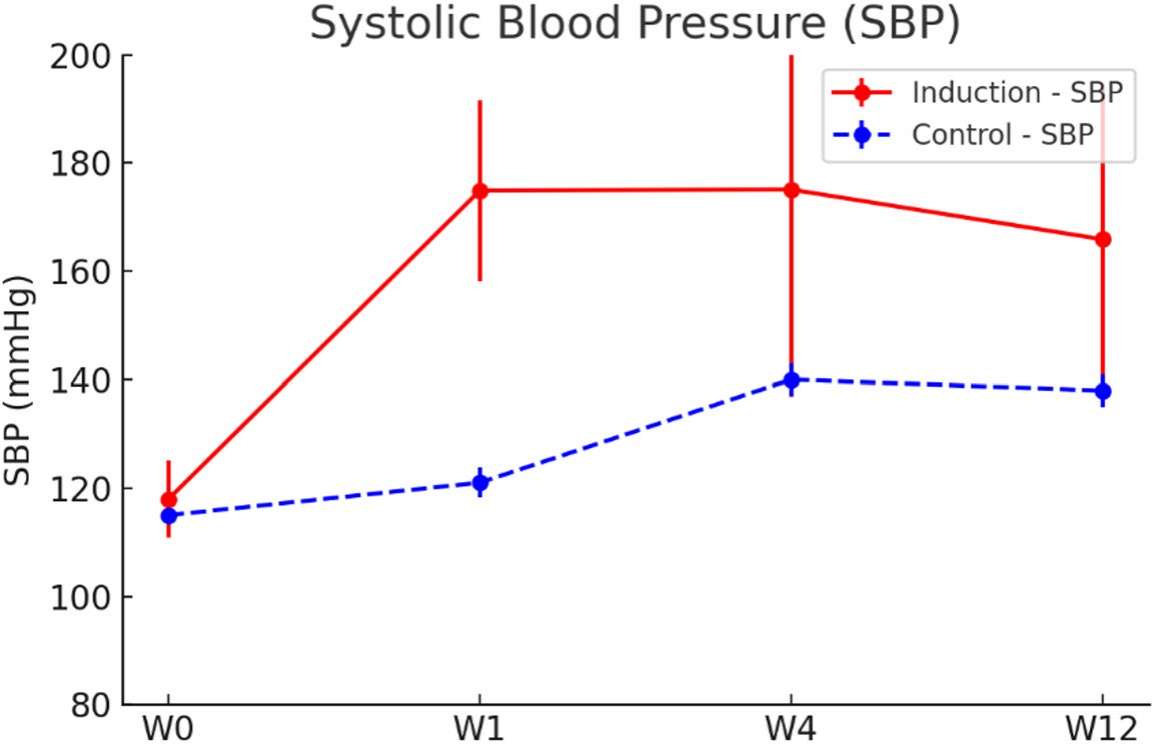
Longitudinal changes in systolic blood pressure (SBP) in both induction and control groups. SBP (mmHg) was serially measured at baseline (W0), W1, W4, and W12. The induction group (*n* = 13, red solid line) showed a rapid and sustained increase in SBP, while the control group (*n* = 6, blue dashed line) exhibited a modest elevation. At each time point, SBP values in the induction group were significantly higher than the W0 as determined by one-sample t-tests based on simulated normally distributed data. Error bars represent standard deviation (SD). *p* < 0.01 at all time points.

Following the induction of hypertension and unilateral carotid ligation, a rapid and sustained elevation in blood pressure was observed. In the induction group (*n* = 13), average systolic blood pressure (SBP) significantly increased from a baseline (W0) of 118 ± 7.1 mmHg to 174.9 ± 16.7 mmHg (W1), and remained elevated at W4 (175.1 ± 38.2 mmHg) and W12 (165.9 ± 28.6 mmHg). While both the induction and control groups (*n* = 6) exhibited a general trend of increasing blood pressure over time, the induction group consistently showed significantly higher values at all time points (*p* < 0.01), indicating a distinct hypertensive effect attributable to the surgical intervention (Fig. 1, Supplementary Fig. 1, Supplementary Fig. 2). To assess whether blood pressure at each post-operative time point significantly differed from baseline (W0), one-sample t-tests were performed. Simulated normally distributed data centered on the mean values at each time point were used for statistical comparison against the W0 baseline.

### Morphologic changes in the COW on MR angiography

**Fig. 2:**
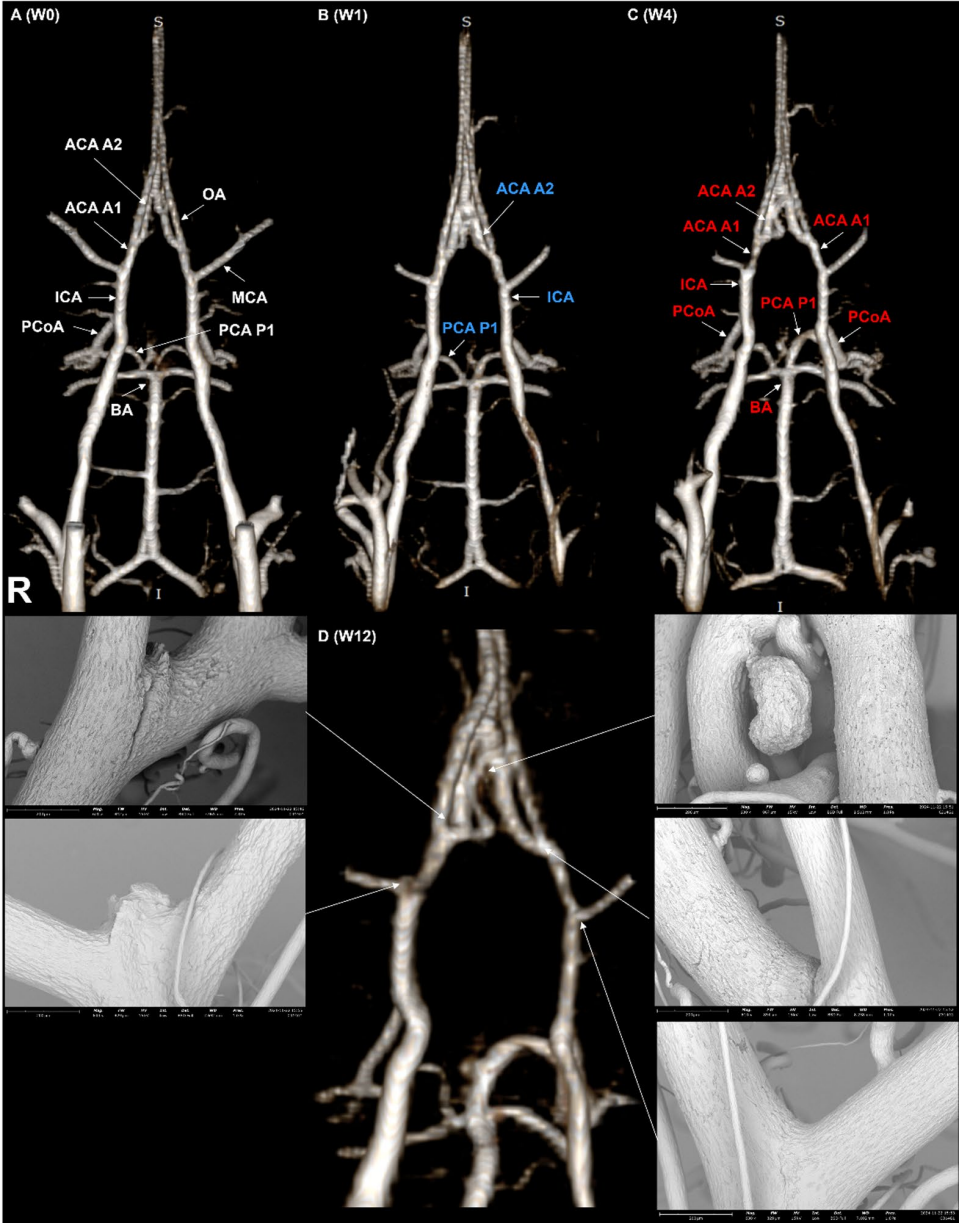
Serial MR angiographic images illustrating longitudinal morphologic changes of the rat COW after aneurysm induction, with corresponding SEM images from the same rat. Representative 3D TOF MRA images are shown at baseline (W0,** A**), W1 (**B**), W4 (**C**), and W12 (**D**). (**A**) The baseline (W0) image depicts anatomical labeling of the vascular segments analyzed: ICA (internal carotid artery), MCA (middle cerebral artery), ACA A1 (proximal segment of the anterior cerebral artery), ACA A2 (distal segment of the anterior cerebral artery), PCA P1 (proximal segment of posterior cerebral artery), PCoA (posterior communicating artery), BA (basilar artery), and OA (olfactory artery). (**B**) The W1 image demonstrates general morphology of the rat COW for reference. Arterial segments shown in blue represent locations exhibiting diameter reductions at W12 across the cohort. (**C**) The W4 image highlights segments in red that showed significant diameter increases compared to baseline (W0), predominantly in the right ICA, left PCA P1, and PCoA. (**D**) At W12, representative postmortem SEM images from the same rat confirm the development of aneurysms at specific sites: right ACA–OA bifurcation (left SEM), right ICA–MCA bifurcation (middle SEM), and right ACA A2 segment (upper right SEM). Two lower right SEM images display normal arteries without aneurysmal lesions for comparison. Note: The color-coded segments indicate general trends observed across the entire cohort and do not reflect site-specific changes in the individual rat depicted here. Statistical analyses were conducted using a linear mixed-effects model to account for repeated measurements. Statistical significance was defined as *p* < 0.05.

At baseline (W0), no significant left-right diameter differences were observed across all arterial segments in either the induction group (*n* = 13) or the control group (*n* = 6), confirming symmetrical COW morphology prior to the surgery (Supplementary Table 1). In the induction group (*n* = 13), serial MR imaging over the 12-week follow-up period quantitatively demonstrated progressive and asymmetric morphologic changes in the COW. All group comparisons and time-based analyses were performed using linear mixed-effects models. A linear mixed-effects model was fitted with time (imaging time points), side (right and left), and their interaction (time × side) as fixed effects, and a random intercept for each rat to account for repeated measurements within subjects. The right internal carotid arteries (ICA) consistently exhibited a significantly larger diameter than the left ICA from W1 onward (*P* < 0.001 at all timepoints), indicating right-sided enlargement following left carotid ligation. Similarly, the left posterior cerebral arteries (PCA) at the P1 segment showed a significantly greater diameter than the right PCA P1 at every timepoint (*P* < 0.001), reflecting compensatory remodeling on the contralateral posterior circulation. These findings suggest that unilateral carotid ligation led to asymmetric vascular adaptation, particularly in the ICA and PCA P1 segments. (Fig. 2)

**Fig. 3:**
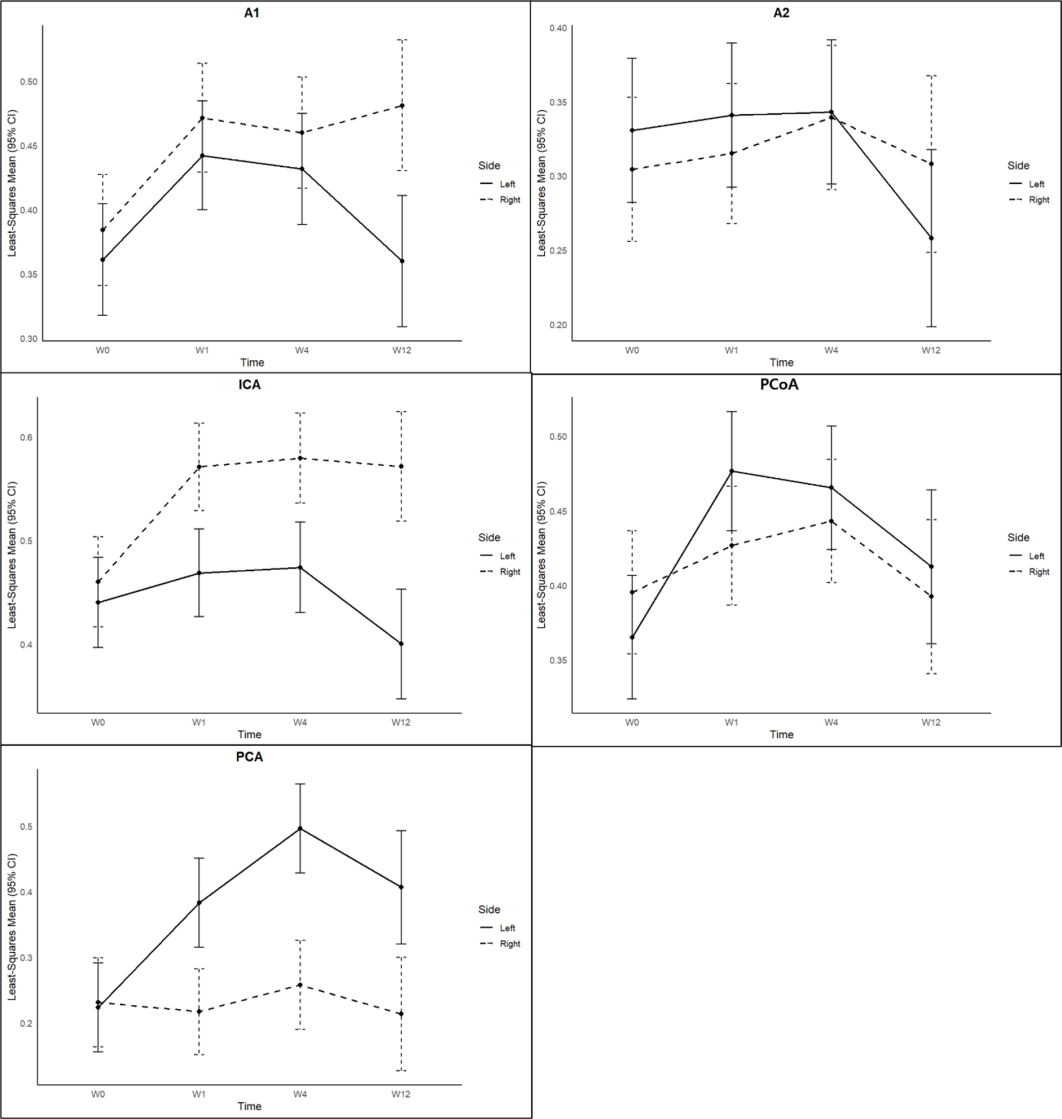
Serial changes in vessel diameter of each segment of the COW in the induction group. Data represent mean diameters of arterial segments on the ligated side (left, solid lines) and the non-ligated side (right, dashed lines). Notable asymmetric vascular remodeling was evident, with significant and progressive diameter enlargement observed in the right ICA (*P* < 0.001) and ACA A1 segments (*P* < 0.05), as well as early and sustained dilation in the left PCA P1 (*P* < 0.001) and PCoA segments (*P* < 0.05). Least-square means and 95% confidence intervals were calculated using linear mixed-effects models with Time, Side, and their interaction as fixed effects, and a random intercept for each rat to account for repeated measurements. Statistical significance was determined by linear mixed-effects model analysis (two-tailed alpha level < 0.05, *n* = 13 animals).

When comparing the induction group (*n* = 13) to the control group (*n* = 6), several arterial segments exhibited statistically significant effects with respect to group, time, and group-by-time interaction. Notably, the right anterior cerebral arteries (ACA) at the A1 segment showed a progressive increase in diameter over time exclusively in the induction group, resulting in significant group and interaction effects (*P* < 0.05). The diameter of the left posterior communicating arteries (PCoA) increased markedly from 0.37 ± 0.04 mm at W0 to 0.48 ± 0.09 mm at W1, and remained elevated thereafter, yielding significant main effects for group and time, as well as their interaction. Similarly, the right ICA displayed significant group and interaction effects, with its diameter progressively increasing from 0.47 ± 0.07 mm at W0 to 0.58 ± 0.03 mm at W12. These findings indicate that surgery-induced vascular remodeling affected both anterior and posterior regions of the COW (Fig. 3).

The tortuosity index (TI) of the left side progressively increased from 1.57 ± 0.08 baseline to 2.12 ± 0.32 at W12, with significant group, time, and interaction effects (*P* < 0.05), reflecting enhanced vascular tortuosity on the ligated side which was the left. In contrast, the right TI also increased but to a lesser extent (from 1.55 ± 0.08 to 1.96 ± 0.23), indicating asymmetric tortuous remodeling. No significant morphologic changes were observed in the control group across any timepoints (Supplementary Table 2). All statistical analyses were performed using a two-tailed significance threshold of α = 0.05.

### Analysis of aneurysm-related findings on MR imaging and SEM

Longitudinal MR imaging suggested AREs in 6 rats (including SAH without a demonstrable aneurysm) out of 13 rats (46.2%) in the aneurysm induction group^7^. Among these, focal arterial protrusions suggestive of unruptured aneurysms were detected in 3 rats. Additionally, SAH was identified in 3 rats, of which 2 exhibited ruptured aneurysms and 1 had SAH without an identifiable aneurysm.

However, SEM and supplementary histologic analysis, serving as the gold standard, revealed AREs in 8 out of 13 rats in the aneurysm induction group, demonstrating a significant discrepancy compared to MR imaging findings. Among these 8 rats, 6 exhibited a single aneurysm, 1 presented with SAH without a demonstrable aneurysm, and 1 rat developed multiple aneurysms, showing three distinct lesions.

**Fig. 4:**
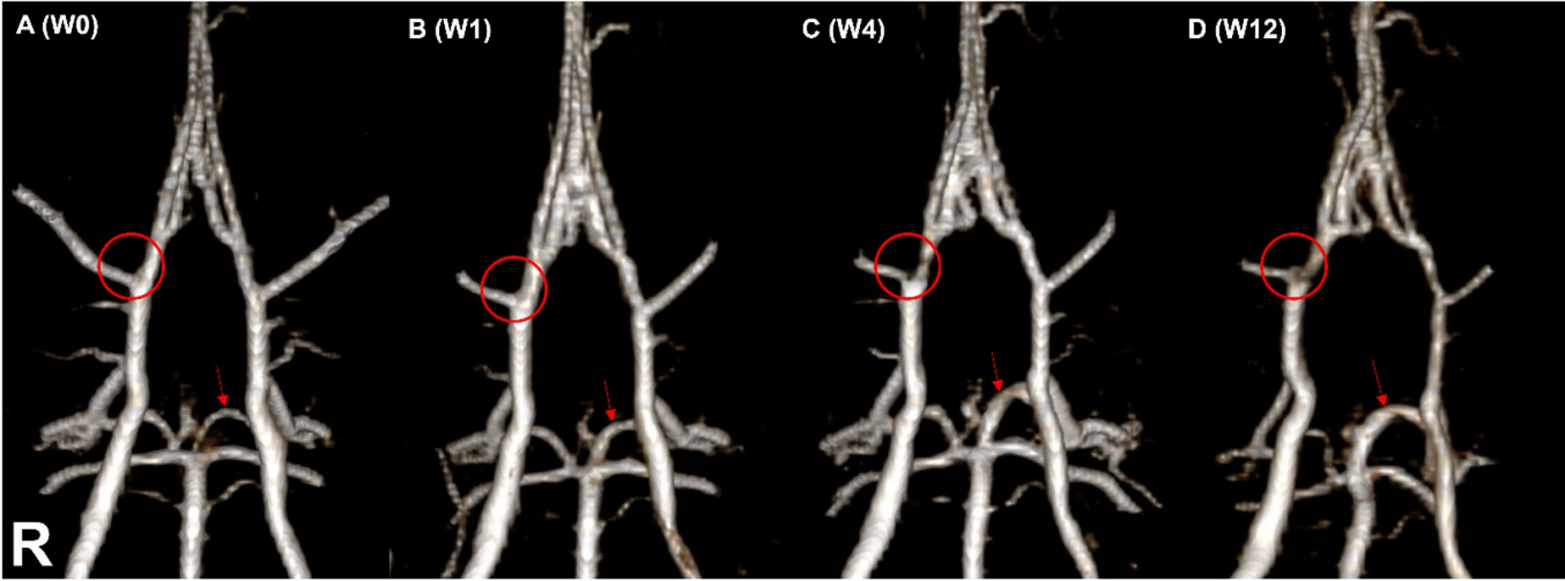
Serial MRA–based detection of small cerebral aneurysms not easily identifiable on single-timepoint imaging. (**A**) Baseline (W0) image with no visible aneurysmal lesion at the ICA–MCA bifurcation. (**B, C**) At W1 and W4, no apparent aneurysmal changes were identified. (**D**) At W12, a newly formed saccular aneurysm (red circle) is evident at the bifurcation. Additionally, progressive dilatation of the left PCA P1 segment (red arrows) was observed throughout the follow-up period, nearly doubling in diameter. Increased vascular tortuosity of the entire COW was also noted from W0 to W12.

**Fig. 5:**
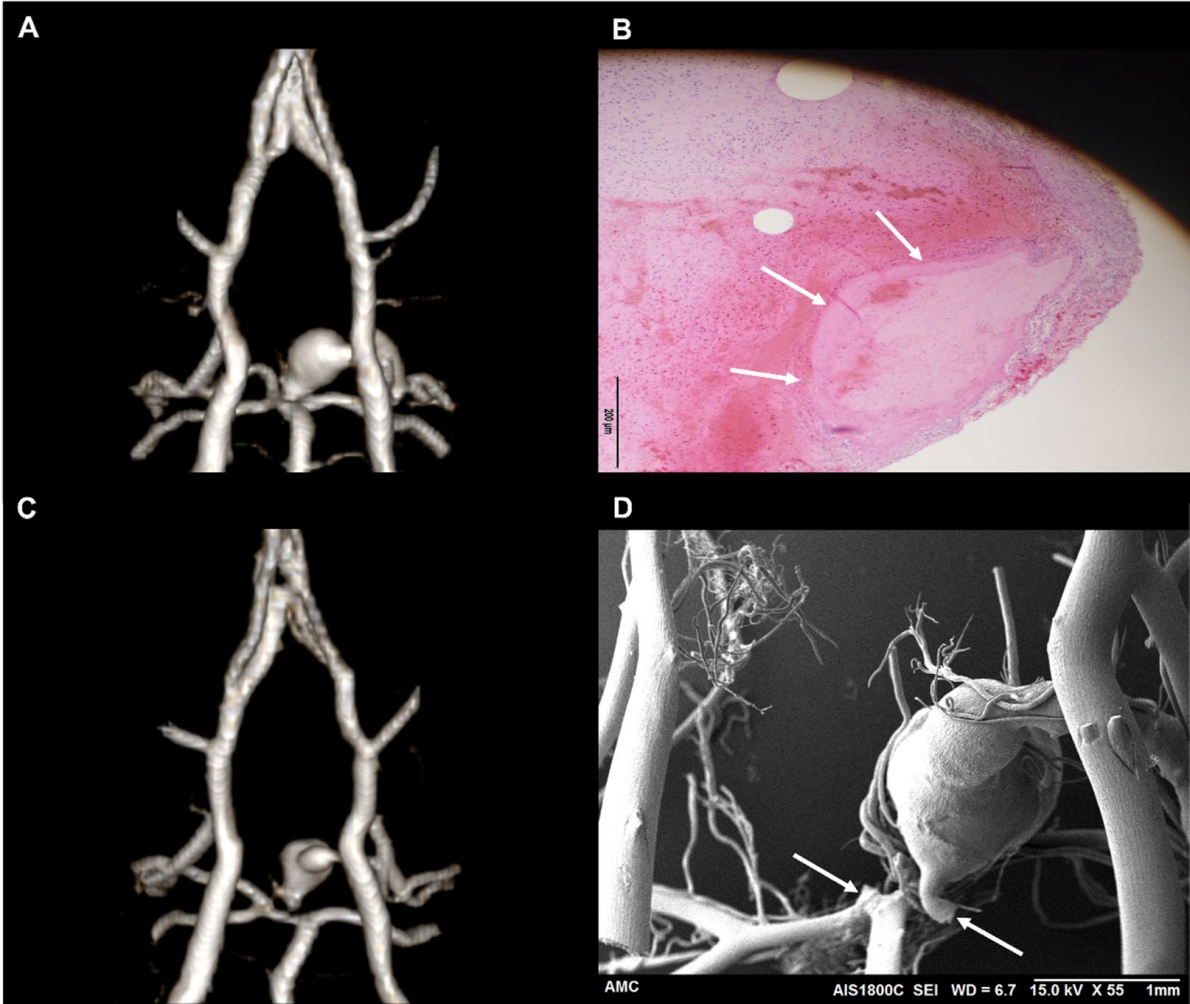
MRA, SEM, and histologic findings of two cases of ruptured PCA P1 aneurysms. (**A**) 3D TOF-MRA from the first case demonstrating a markedly enlarged fusiform aneurysm at the left PCA P1 segment. Due to extensive intracranial hemorrhage caused by aneurysm rupture during brain extraction, corrosion casting was not feasible in this animal. (**B**) Histologic section (H&E staining) from the first case showing the aneurysmal lesion (white arrow). (**C**) 3D TOF-MRA from the second case also exhibiting a large fusiform aneurysm at the left PCA P1 segment. (**D**) Corresponding SEM image from the second case revealing prominent endothelial surface irregularities and morphological changes at the aneurysm site. White arrows indicate areas where accidental breakage occurred during removal of the corrosion cast. These findings highlight the formation and rupture potential of fusiform aneurysms in the posterior circulation induced by hemodynamic stress.

Although the number of animals with AREs was 8, our study aimed to evaluate the diagnostic performance of longitudinal MR imaging at the level of each individual ARE, rather than at the animal level. Therefore, in cases with multiple aneurysms, each lesion was counted as a separate event for analysis. To calculate the diagnostic performance of MR imaging, we defined the total number of events (N) as 15, including 10 confirmed AREs and 5 ARE-negative animals, each considered as one negative event. In addition, the case with SAH but without a demonstrable aneurysm was included as a single positive event due to its clinical relevance. Based on this lesion-based approach, the 10 SEM or histology-confirmed AREs were classified as follows: (1) Unruptured aneurysms detected by both MR and SEM (*n* = 1, Fig. 4D), (2) Unruptured aneurysms detected only by SEM (*n* = 6), (3) Ruptured aneurysms detected on both MR and SEM/histology (*n* = 2, Fig. 5), (4) SAH without a demonstrable aneurysm, identified on both MR and SEM (*n* = 1). (Table 1)

**Table 1:** Summary of the 10 individual lesion-based AREs, 2 false-positive lesions suggested on MR imaging, and 5 ARE-negative cases.

Types of SEM/histology-confirmed aneurysm (N = 10)	Location	Shape	Timing (week)	Diameter on MR (mm)	Diameter on SEM (mm)	Concordance with MR imaging
Unruptured aneurysms detected by both MR and SEM (n = 1)	Right ICA-MCA bifurcation	saccular	12	0.22	0.21	Yes
Unruptured aneurysms detected only by SEM (n = 6)	Left ACA-OA origin	saccular	12	NA	0.03	No
	Right ACA A2 segment	saccular	12	NA	0.07	No
	Right ICA-MCA bifurcation	saccular	12	NA	0.07	No
	Right ICA-MCA bifurcation	saccular	12	NA	0.08	No
	Right ACA-OA origin	saccular	12	NA	0.10	No
	Right ACA-OA origin	saccular	12	NA	0.10	No
Ruptured aneurysm detected on both MR and SEM (n = 2)	Left PCA P1 segment	fusiform	4	1.45	NA	Yes
	Left PCA P1 segment	fusiform	*6	0.55	1.34	Yes
Unidentified aneurysm with SAH on both MR and SEM (n = 1)	NA	NA	11	NA	NA	Yes
False-positive aneurysm suggested on MR (n = 2)	Location	Shape	Timing (Week)	Diameter on MR (mm)	Max. Diameter (mm)	Concordance with SEM
	Left PCA P1 segment	saccular	12	0.30	0.30	No
	Right ACA A2 junction	saccular	12	0.35	0.25	No
Rats with no ARE (n = 5)	NA	NA	**12	NA	NA	NA

*This subject showed no abnormalities up to week 4, but began to exhibit neurological deficits after week 5, prompting early MR imaging and subsequent corrosion casting at week 6.

** Except 2 rats, died (7 and 11 weeks) early due to unknown etiology.

Specifically, lesion sites were labeled using the format “Parent Vessel–Branch or Anatomical Landmark”, enabling intuitive and consistent designation of aneurysm-prone regions^9^. For example, aneurysms arising at the origin of the olfactory artery from the anterior cerebral artery (ACA) were denoted as “ACA–OA origin”, while those located at the ICA bifurcation into the middle cerebral artery (MCA) were labeled “ICA–MCA bifurcation.” Similarly, junctional and segmental lesions were described as “ACA–A1–A2 junction”, “PCA–P1 mid-segment,” or “ACA–A2 distal segment,” as appropriate. This nomenclature was uniformly applied throughout the study to describe both suspected and confirmed aneurysm sites.

The results revealed 6 false negatives and 2 false positives on MR imaging. These findings are summarized in the confusion matrix shown in the Table 2. Based on lesion-level analysis, MR imaging demonstrated a sensitivity of 40% (95% confidence intervals (CI): 16.8% – 68.7%, 4 true-positive out of 10 confirmed lesions), a specificity of 60.0% (95% CI: 23.1% – 88.2%, 3 true negatives out of 5 MR-negative cases), a positive predictive value (PPV) of 66.7% (95% CI: 30.0% – 90.3%, 4 true positives out of 6 MR-positive lesions), a negative predictive value (NPV) of 33.3% (95% CI: 12.1% – 64.6%, 3 true negatives out of 9 MR-negative lesions), and an overall diagnostic accuracy of 46.7% (95% CI: 24.8% – 69.9%, 7 correct classifications out of 15 total cases). Diagnostic performance metrics including sensitivity, specificity, PPV, NPV, and accuracy were calculated using standard definitions based on the confusion matrix. (Table 2)

**Table 2:** Confusion matrix showing the diagnostic performance of the MR imaging for the AREs.

Lesion-level	ARE confirmed on SEM	No ARE on SEM	Total
ARE on MR	4	2	6
No ARE on MR	6	3	9
Total	10	5	15

AREs detected on MR imaging were compared against those confirmed on SEM. Sensitivity, specificity, PPV, NPV, and overall accuracy were calculated from this matrix. All diagnostic performance metrics were calculated along with 95% CI using the Wilson method. Data represent 15 animals.

The median diameter of aneurysms confirmed by SEM was 0.26 mm (range, 0.03–1.34 mm). These measurements were obtained from SEM images in all but one case. In that exceptional case—a large, ruptured fusiform aneurysm in the left PCA P1segment—we were unable to obtain a corrosion casting or histological specimen due to the massive extent vague margin of the aneurysm sac of the ruptured lesion. Consequently, the aneurysm diameter in that case was measured using MR imaging data acquired prior to the rupture, which showed a diameter of 1.45 mm. MR-detected aneurysms (*n* = 5) were larger, with a median size of 0.35 mm (range, 0.22–1.45 mm). MR false-negative lesions were generally smaller when measured on SEM images (≤ 0.1 mm) occurring at left ACA-OA origin (*n* = 2), right ICA-MCA bifurcation (*n* = 2), and right A2 segments (*n* = 1). MR false-positive cases, likely reflecting imaging artifacts or marked tortuosity of the dilated arteries, were found at left PCA P1 segment (*n* = 1) and the right A2 junction (*n* = 1).

Among the 10 confirmed AREs, stratification by rupture status revealed a distinct morphological pattern. All unruptured aneurysms were small saccular lesions with a maximal diameter ≤ 0.1 mm. In contrast, aside from one lesion associated with SAH in which no aneurysm could be identified by either imaging or corrosion casting, the remaining two ruptured aneurysms demonstrated a fusiform morphology as a result of rapid dilation of the PCA P1 segment, and were notably large, with maximal diameters exceeding 1 mm.

## Discussion

In this study, we demonstrated the technical feasibility of performing repeat longitudinal MR imaging, including MRA in experiment using the Hashimoto rat cerebral aneurysm model. Notably, arterial changes in the COW were already evident as early as one week after aneurysm induction. These changes progressed in a segment-specific manner, with clear asymmetry observed between the left and right hemispheres. While MRA allowed the detection of aneurysms at multiple time points, a substantial number of aneurysms confirmed by corrosion casting were too small to be visualized on MR images, underscoring the current limitations in spatial resolution.

Previous studies have explored cerebral aneurysm formation in mouse or rat models using high-field preclinical MR imaging systems^6,8^, and the diagnostic potential of this modality has been suggested^10^. Compared to conventional small animal aneurysm models, the ability to visualize aneurysms in vivo was considered a promising advancement^7^. However, our current study revealed that many aneurysms formed in the Hashimoto model were exceedingly small in size. Despite recent improvements in preclinical TOF-MRA technology, we found that the spatial resolution of current imaging protocols remains insufficient to capture these tiny lesions. In this study, the spatial resolution of TOF-MRA was calculated to be approximately 0.146 × 0.146 × 0.146 mm³ based on the applied field of view and matrix size. Notably, among the six false-negative aneurysms identified in the induction group, the largest lesion confirmed by SEM measured only 0.10 mm in diameter. This strongly suggests that the false negatives were attributable to the inherent limitations in spatial resolution of the current imaging protocol^7^.

Nevertheless, we believe that longitudinal repeat MR imaging remains a valuable tool in preclinical research^6^. In cases where the aneurysms were large enough to be detected by MRA, we were able to monitor their formation and morphological evolution over time. Furthermore, even in animals without aneurysm development, serial MR imaging enabled the assessment of hemodynamic stress-induced morphologic changes in the COW—particularly in terms of arterial diameter and tortuosity^11^. These observations underscore the utility of this approach for studying in vivo vascular remodeling and aneurysm pathogenesis in induced cerebral aneurysm models.

In addition to TOF-MRA, supplementary acquisition of T2-weighted imaging (T2WI) and BBI proved useful in the assessment of vascular changes and the detection of aneurysms^6^, as well as in the radiologic diagnosis of SAH. Notably, BBI was particularly valuable in distinguishing true segmental narrowing from artifacts commonly seen on TOF-MRA—especially after week 4—thus complementing the limitations of flow-dependent imaging. However, given the substantially longer acquisition time required for BBI, its routine inclusion in longitudinal imaging protocols warrants careful consideration^6^.

In this study, we observed substantial morphologic alterations in the COW following hemodynamic modulation. These changes were attributed not only to the globally increased arterial stress induced by hypertension, but also to the asymmetrical hemodynamic shifts caused by unilateral left common carotid artery (CCA) ligation. In the anterior circulation, we noted enlargement of the ACA segments and the formation of a collateral channel between the bilateral ACA A2 segments, functioning similarly to a human anterior communicating artery (ACoA). This channel, which was not visible at baseline, became clearly discernible by week 1 in most animals. Although the rat COW has traditionally been described as lacking a well-developed ACoA, our findings suggest that a fine, previously inapparent communicating channel may exist and become functional under altered hemodynamic conditions. This observation is consistent with the anatomical description by Lee RM^12^, who noted the ACoA in rats as being poorly developed or nearly invisible. Additionally, at the terminal portion of the ACA A2 segments, we frequently observed the formation of a short azygos ACA, which branched into small cortical arteries supplying both cerebral hemispheres. Such vascular reorganization would be difficult to detect without longitudinal MR imaging in a normal rat model.

In the posterior circulation, we observed a consistent increase in flow from the basilar artery to the left PCA following left CCA ligation, leading to a marked enlargement of the left PCA P1 segment as early as week 1 (Fig. 4). Notably, in two animals, the left P1 segment dilated beyond 1 mm, forming large fusiform aneurysms that subsequently ruptured. This represents a novel observation not previously reported in similar models. In nearly all animals, the left P1 diameter at week 1 was approximately twice that of baseline (W0); however, in these two cases, the expansion was even more prominent, culminating in fusiform aneurysm formation and rupture^13^. Interestingly, while the left P1 segment showed significant dilatation, the contralateral right P1 segment remained unchanged in diameter throughout the study. This suggests that bilateral comparison of PCA P1 segments could serve as a good quantitative metric sites for evaluating vessel-specific responses to hemodynamic stress^14^. Although histologic evaluation of this asymmetry was not performed in this study, MRA clearly demonstrated a consistent and significant morphologic difference between the two sides.

In addition to the PCA P1 segment, the ICA–MCA bifurcation was another critical site of observation. Notably, this was the only location in our study where an unruptured saccular aneurysm was simultaneously identified on both MR imaging and SEM (Fig. 4). The lesion appeared relatively large and morphologically distinct, which made it visible on MR images. However, the aneurysmal contour was still subtle on MRA and might have been missed without prior baseline imaging. This reinforces the diagnostic value of repeat longitudinal imaging in improving detection sensitivity, particularly for small or marginal lesions.

**Fig. 6:**
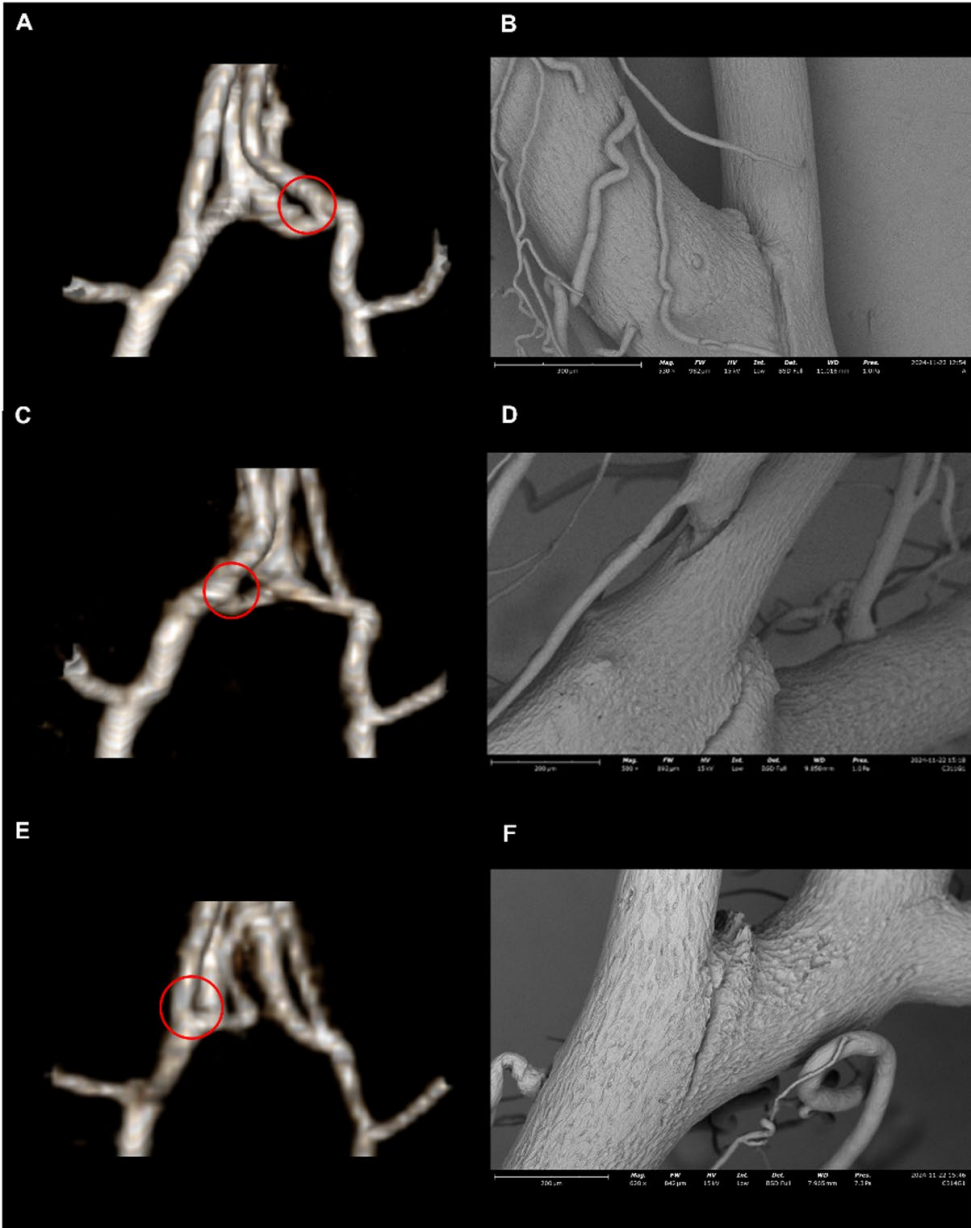
Microaneurysms at the ACA–OA origin confirmed by SEM but undetectable by MRA. Three representative cases of microaneurysms at the ACA–OA origin (case 1: **A–B**, left; case 2: **C–D**, right; case 3: **E–F**, right). MR images (**A, C, E**) failed to detect these lesions due to their small size, whereas SEM images (**B, D, F**) clearly confirmed them. Red circles indicate corresponding aneurysm locations confirmed by SEM. These lesions appeared as small spike-like protrusions rather than typical saccular aneurysms. Unlike prior reports from the Hashimoto rat model, aneurysms at the ACA–OA origin were relatively uncommon in our study, highlighting variability in this model.

Excluding the case of SAH without an identifiable aneurysm, analysis of the remaining nine confirmed aneurysms revealed that most were located at arterial bifurcations (6 out 9 aneurysm). Two large lesions on the left PCA P1 segment were classified as fusiform aneurysms, while one very small lesion at the right ACA A2 segment appeared to be a lateral wall aneurysm. Interestingly, unlike prior studies based on the Hashimoto model which frequently reported aneurysm formation at the right ACA–OA bifurcation^4,15,16^, our study identified only two such cases at the right ACA–OA and one at the left ACA–OA bifurcation. Given this distribution, these bifurcation sites were not the most prevalent locations in our cohort. Furthermore, the morphology of the aneurysms at the ACA–OA bifurcation was distinct from typical human saccular aneurysms, instead appearing as very tiny, spike-like protrusions^15^ (Figs. 2D and 6), raising questions about the appropriateness of using this site as a primary region of interest in induced aneurysm models^16^.

A notable limitation highlighted in this study was the presence of two false-positive aneurysm findings on MR imaging, which underscores a technical constraint in small animal preclinical models using MR. The first case involved a suspected aneurysm at the ACA A2 segment. Upon closer analysis, it was determined that this finding was likely due to an enlarged and tortuous arterial segment representing the fine channel corresponding to the ACoA, which can often mimic aneurysmal morphology. Such tortuous configurations were frequently observed in other animals as well and may lead to misinterpretation, necessitating cautious image evaluation^17^. The second case involved a focal lesion at the proximal portion of the PCA P1 segment extending in a medial superior direction. However, SEM imaging revealed that the finding corresponded to overlapping conglomerated perforating arteries, rather than a true aneurysm. This again highlights the limitations imposed by the spatial resolution of MR imaging. Although future advancements in imaging resolution may help mitigate false-positive interpretations, these examples underscore the current limitations and the need for careful anatomical correlation.

In addition to the aforementioned limitations, we recognized several study-specific constraints in the current investigation. First, although many interesting findings were observed, the small sample size of rats used in this study limits the generalizability of our results. In particular, the two striking fusiform aneurysms identified in the left PCA P1 segment were not previously observed in our pilot studies, highlighting the need for caution when interpreting these rare findings although a recent study has also showed similar lesions in the P1 segment^18^. Second, due to the nature of this in vivo longitudinal imaging study, we were unable to perform histological correlation for the observed vascular changes, which limits our understanding of the underlying pathology. Third, due to the absence of dedicated post-processing software tailored for small animal TOF MRA, we utilized clinical software not originally designed for such small-scale data, which may have introduced processing limitations^19^. Fourth, aneurysm diagnosis was based on volume-rendered reconstruction images; however, the reconstruction conditions could not be standardized, and were adjusted based on operator experience. Although we attempted to minimize variability by using the Full Width Half Maximum (FWHM) method for diameter measurements, this remains a technical limitation^20^. Fifth, the current study focused primarily on the COW, which may have led to under-detection of peripheral cortical arterial lesions. This limitation is exemplified by one case in which SAH was evident, but no aneurysm could be identified within the imaged territory. Notably, similar concerns have been raised in previous animal studies utilizing MRA, where SAH occurred without detectable aneurysms^21^, likely due to the limited spatial coverage and resolution of standard imaging protocols. Therefore, we should consider strategies to overcome this limitation, recognizing that aneurysmal rupture may occur in distal cerebral arteries beyond the COW, and that comprehensive vascular assessment should account for such possibilities during image interpretation. Finally, although all subjects in this study were female rats, oophorectomy was not performed. As hormonal status may influence aneurysm formation, this limits direct comparison with other studies using oophorectomized models. Future studies using oophorectomized female rats may provide additional insights into hormonal contributions to aneurysm pathogenesis^22^.

High-resolution preclinical MR imaging was found to be suitable for longitudinal assessment of COW morphology in the Hashimoto rat cerebral aneurysm model. While the current imaging resolution imposed clear limitations in detecting aneurysms smaller than the spatial threshold, the model nonetheless offers valuable insight into the dynamic vascular changes associated with aneurysm formation, growth, and rupture risk. With continued refinement of imaging protocols and analytic tools, this platform may further advance our understanding of intracranial aneurysm pathophysiology and support the evaluation of therapeutic strategies.

## Methods

This study was conducted with the approval of the local Institutional Animal Care and Use Committee of the Asan Institute for Life Sciences, Asan Medical Center, Seoul, Republic of Korea (IACUC, 2023-20-262). All animal procedures were performed in accordance with relevant institutional guidelines, national regulations, and are reported in compliance with the ARRIVE (Animal Research: Reporting of In Vivo Experiments) guidelines. The experimental design included the development of a rat model, serial MR image acquisition, validation using corrosion casting and histologic analysis, as well as subsequent image interpretation and statistical analysis, as outlined below.

## Animal model creation

Female Sprague-Dawley rats, aged 8 weeks, were assigned to either a control group (*n* = 6) or an experimental aneurysm induction group (*n* = 15). All Sprague-Dawley rats were obtained from JA BIO, Inc. (Suwon-si, Gyeonggi-do, Republic of Korea). The induction protocol involved unilateral ligation of the left CCA and the right renal artery (RA) under isoflurane anesthesia (3% for induction, 2% for maintenance)^16,23^. For the CCA ligation, an incision was made at the midline of the neck, and the surrounding fat tissue was gently dissected to expose the vascular structure. The CCA was identified and ligated with Black Silk 3 − 0 suture. The procedure was followed by right lateral flank incision for the right RA ligation. Under the microscopic manipulation, the right main renal artery was identified and ligated using the same suture material. Post-surgery, 1 mL of antibiotics (Gentamicin Injection; 1 ml, Shin Poong Pharmaceutical, Seoul, Korea) and analgesics (Ketocin Inj; 1 ml, Myung Moon Pharmaceutical, Seoul, Korea) were administered via intraperitoneal injection. Following the injection, the animals were placed on a heat pad set at 39 °C and simultaneously underwent infrared therapy for at least 5 min. Following surgery, the rats were provided free access to a high-sodium diet (8% NaCl) supplemented with 0.12% β-aminopropionitrile (BAPN), a known lysyl oxidase inhibitor^24^. Except for animals that died due to AREs before the completion of imaging protocols, all surviving animals underwent vascular corrosion casting. For animals undergoing vascular corrosion casting, deep anesthesia was induced with isoflurane (5% for induction, 2–3% for maintenance) using a vaporizer. After confirmation of complete anesthesia, a thoracotomy was performed to expose the heart, and euthanasia was achieved by transcardial perfusion with 100 ml of a heparin/phosphate-buffered saline solution (20 U/ml), resulting in exsanguination and cessation of cardiac activity. Subsequently, Batson’s #17 casting compound (Polysciences, Inc., Warrington, PA, USA) was infused to create vascular casts.

Surgical success was defined as the successful execution of the planned vascular manipulations and the animal’s survival with adequate clinical condition to undergo scheduled MR imaging at week 1 postoperatively. Animals were considered surgical failures if they experienced intraoperative or immediate postoperative mortality, or survived the surgery but did not maintain sufficient clinical condition to complete the scheduled MR imaging session at W1. The control animals underwent sham operations without vascular manipulation and were maintained on a standard diet.

### MR imaging protocol

In vivo MR imaging was performed using a 7.0 Tesla preclinical MRI system (Bruker BioSpin, Ettlingen, Germany) equipped with a dedicated rat coil. Imaging sessions were scheduled at baseline W0, and at W1, W4, and W12 post-operatively. Each session included a comprehensive protocol incorporating TOF-MRA, T2WI, and proton-density BBI.

T2-weighted axial and coronal images were acquired to assess anatomical structures with the following parameters: an echo time (TE) of 66 ms, a repetition time (TR) of 5250 ms, a flip angle of 16°, a slice thickness of 1 mm, a field of view (FOV) of 28 × 28 mm2, the matrix size of 256 × 256, and a scan time of 2 min per sequence.

The parameters for 3D TOF-MRA included a TE of 2 ms, a TR of 12 ms, a flip angle of 20°, a 3D volume thickness of 28 mm on coronal orientation, a field of view of 28 × 28 × 28 mm3, a matrix size of 192 × 192 × 192, and a scan time of 12 min. The resulting isotropic spatial resolution of the 3D TOF-MRA was approximately 0.146 mm, derived from a 28 mm³ FOV and a 192³ matrix.

The parameters for BBI were performed to visualize the vascular outlines while suppressing the flowing blood signal. T2-weighted axial imaging was utilized with the following parameters: an TE of 7.5 ms, a TR of 1900 ms, an echo spacing of 7.5 ms, a turbo factor of 4, and 8 averages. The FOV was set to 28 × 28 mm2 with a matrix size of 384 × 384, providing high spatial resolution. The slice thickness was set at 0.3 mm, and a total of 30 axial slices were acquired per package, covering a slab thickness of 27 mm. The resulting spatial resolution of BBI was approximately 0.073 mm in-plane and 0.3 mm through-plane. The inversion time was 700 ms, and a calculated pulse shape was used along with the “Auto Slice Spoiler” function to enhance blood signal suppression. The scan time was approximately 18 min.

Animals exhibiting signs of neurological deficit or unexplained mortality underwent immediate imaging using the routine protocol whenever possible. In cases where animal condition precluded completion of the full protocol, imaging was limited to TOF-MRA only. For animals found dead, imaging was restricted to T2WI alone before autopsy, to assess the presence of intracranial lesions including SAH.

### Blood pressure monitoring

Blood pressure measurements were performed in all animals (induction group: *n* = 13, control group: *n* = 6) using a non-invasive tail-cuff plethysmography system (CODA 8; Kent Scientific, Torrington, CT, USA). Because MR imaging required general anesthesia, and tail-cuff blood pressure measurement demands the animal to be awake and acclimated to restraint, it was impractical to conduct both procedures on the same day. Therefore, blood pressure recordings were conducted either one day before or one day after each MR imaging session to avoid stress and ensure measurement accuracy under awake conditions. Each measurement consisted of at least five replicates, and SBP, diastolic blood pressure (DBP), and mean arterial pressure (MAP) were recorded. For each time point, the average of valid readings was used in the analysis.

### Longitudinal analysis of cerebral arterial morphology

Quantitative morphometric analysis was performed on the COW, focusing on the measurement of arterial diameters across 11 predefined segments. These included bilateral ICA, ACA at the A1 and A2 segments, PCA at the P1 segment, and PCoA, as well as the distal basilar artery (BA). Specifically, the following segments were measured: right ICA, right ACA A1, right ACA A2, right PCA P1, right PCoA, left ICA, left ACA A1, left ACA A2, left PCA P1, left PCoA, and the distal BA (Fig. 2).

Vessel diameters were derived from TOF-MRA datasets using the VasoMetrics macro (VasoMetrics; Konnor McDowell at the Seattle Children’s Research Institute, Seattle, WA, USA)^25^ based on the FWHM algorithm implemented in ImageJ (National Institutes of Health and the Laboratory for Optical and Computational Instrumentation, Bethesda, MD, USA).

In the evaluation of the arterial tortuosity, we focused on the COW arterial tortuosity change. Before calculating the TI, we defined two anatomical landmarks: the ACA point, located at the midline where the bilateral ACAs face each other—corresponding to the junction between ACA A1 and A2 segments—and the BA point, defined at the site where the BA bifurcates into the bilateral PCAs. Using these two reference points, three measurement paths were established: a right-sided curved line drawn along the centerline of the right side of the COW, a left-sided curved line along the left side, and a straight line directly connecting the ACA point and the BA point. TI of each side of the COW could be calculated by dividing the curved-line length with the straight-line length. We measured their length in 3D space using the 3D Slicer. As a result, we could get the TI’s of both sides of the COW. We regarded increase of the TI as increase of the arterial tortuosity^26,27^.

### Vascular corrosion casting for SEM

Rats were anesthetized via inhalation of a 2% isoflurane mixture using a vaporizer. Following full anesthesia, a thoracotomy was performed to expose the heart. A 19-gauge plastic cannula (1.25 inches in length) was inserted into the left ventricle. Perfusion was conducted through the left ventricle using 100 ml of a 1:9 mixture of heparin/phosphate-buffered saline (20 U/ml), until the outflow from the right atrium appeared sufficiently diluted. Prior to complete clearance of blood, the corrosion casting compound (Batson’s #17, Polysciences, Inc., Warrington, PA, USA) was prepared by mixing blue dye, 20 ml of base solution, 3 ml of catalyst, and 3 drops of promoter. Approximately 10 ml of the prepared compound was slowly infused. Adequate perfusion was confirmed by observing blue discoloration in the eyes and extremities. The entire body was then stored in ice water at 4 °C for 24 h. After fixation, the vascular cast including the brain was carefully dissected. The specimen was subsequently immersed in 10% potassium hydroxide for 24 h to dissolve surrounding soft tissues, rinsed, then immersed again for an additional 24 h. A final rinse completed the preparation for further SEM evaluation^28,29^.

Corrosion cast specimens of rat brains were examined using a scanning electron microscope (SEM; AIS 1800 C, Seron Technologies, Uiwang-si, Korea). SEM evaluation was performed on vascular corrosion casts obtained from the aneurysm induction group, excluding animals that died prematurely or in which corrosion casting could not be performed, resulting in a total of 8 rats. Samples were mounted on aluminum stubs with conductive carbon tape and sputter-coated with a thin layer of gold (30 mA, 240 s) to prevent charging. All specimens were processed to preserve surface morphology and to ensure adequate conductivity for high-resolution imaging. SEM imaging was performed under high vacuum conditions (1 × 10⁻⁴ to 1 × 10⁻⁶ Torr) in the specimen chamber. Imaging parameters, including accelerating voltage and working distance, were adjusted to enhance image resolution and contrast, enabling detailed visualization of the corrosion cast morphology.

### Analyses of AREs on MR imaging and SEM

MIP and 3D volume rendering images of the longitudinal TOF-MRA were assessed for the presence of focal arterial protrusions suggestive of aneurysm formation^30^. In cases with equivocal findings, 2D source images and corresponding BBI images were used for clarification. Aneurysm diagnosis was made based on consensus between two independent observers; any disagreements were resolved by a third reviewer. An aneurysm was defined as any focal protrusion of the arterial wall that was visually identifiable, regardless of the size of the parent vessel.

SEM examination of the corrosion casts served as the gold standard to validate aneurysm presence, morphology, and distribution identified on MR imaging. For animals that died prematurely or when corrosion casting failed, autopsy including craniotomy and hematoxylin and eosin (H&E) staining-based histological examination of the COW was performed.

AREs were classified into four categories:

1. Unruptured aneurysm confirmed by both MR imaging and SEM.
2. Unruptured aneurysm identified only by SEM.
3. Ruptured aneurysm with radiologic evidence of SAH.
4. SAH without detectable aneurysm.

For aneurysm size measurements, sac height was recorded for saccular aneurysms, while the maximal transverse diameter was used for fusiform aneurysms. MR-based measurements were obtained from maximum intensity projection (MIP) reconstructions of TOF-MRA datasets at the final imaging time point. For fusiform aneurysms, optimized MIP views generated from volume-rendered images were used to enhance measurement accuracy. SEM-based measurements were performed by selecting the view (anterior or posterior) that provided the clearest delineation of the aneurysm contour. Each aneurysm was measured three times independently in both MR and SEM datasets, and the average value was recorded.

Anatomical locations were documented individually for all lesions, including cases with multiple aneurysms per animal. Finally, the frequency, characteristics, spatial distribution (location), and temporal evolution of all AREs were comprehensively analyzed based on combined MR imaging, histopathology, and SEM findings. aneurysm locations were described based on a standardized anatomical naming convention that combines the primary vessel name with its relevant branching point or anatomical landmark. Specifically, lesion sites were labeled using the format “Parent Vessel–Branch or Anatomical Landmark”, enabling intuitive and consistent designation of aneurysm-prone regions^9^. For example, aneurysms arising at the origin of the olfactory artery from the anterior cerebral artery (ACA) were denoted as “ACA–OA origin”, while those located at the ICA bifurcation into the middle cerebral artery (MCA) were labeled “ICA–MCA bifurcation.” Similarly, junctional and segmental lesions were described as “ACA–A1–A2 junction”, “PCA–P1 mid-segment,” or “ACA–A2 distal segment,” as appropriate. This nomenclature was uniformly applied throughout the study to describe both suspected and confirmed aneurysm sites.

### Statistics

Statistical analyses were performed to examine time-dependent vascular changes and AREs. Linear mixed-effects models, which account for repeated measures within individual animals were used. Notably, this modeling approach was specifically selected to address the substantial missing data resulting from early mortality in the later stages of the study, as LMMs are particularly robust for analyzing unbalanced datasets without excluding subjects with incomplete follow-up. Fixed effects included time (W0, W1, W4, W12), group (induction vs. control), and their interaction (time × group), while random intercepts were used to control for inter-subject variability. Significant time × group interaction terms were interpreted as evidence of differential vascular remodeling patterns between groups. Post-hoc comparisons were performed using Tukey’s Honestly Significant Difference (HSD) test. Additional subgroup comparisons were made between hemispheres (left vs. right) and between animals with and without AREs. All statistical tests were two-sided, and significance was defined as *p* < 0.05. Statistical analyses were performed using RStudio (version 2025.05.1 + 513; Posit Software, PBC, Boston, MA, USA).

### Use of AI tools

Portions of the Methods section were revised with the assistance of ChatGPT (OpenAI), a large language model, to improve grammar, clarity, and conciseness of the English text. All AI-assisted edits were carefully reviewed and verified by the authors to ensure scientific accuracy and appropriateness.

## Supplementary Information

Below is the link to the electronic supplementary material.

Supplementary Material 1

## Data Availability

The datasets generated and/or analyzed during the current study (including MR and SEM images, vascular measurements, and statistical data) are available from the corresponding author upon reasonable request.

## References

[1] Vlak, M. H. et al. Prevalence of unruptured intracranial aneurysms, with emphasis on sex, age, comorbidity, country, and time period: A systematic review and meta-analysis. *Lancet Neurol.* **10**, 626–636 (2011). 21641282 10.1016/S1474-4422(11)70109-0

[2] Nieuwkamp, D. J. et al. Changes in case fatality of aneurysmal subarachnoid haemorrhage over time, according to age, sex, and region: A meta-analysis. *Lancet Neurol.* **8**, 635–642 (2009). 19501022 10.1016/S1474-4422(09)70126-7

[3] Sutkowska, K. et al. Impact of the transforming growth factor beta (TGF-beta) on brain aneurysm formation and development: A literature review. *Cell. Mol. Neurobiol.* **45**, 46 (2025). 40392340 10.1007/s10571-025-01572-y PMC12092881

[4] Hashimoto, N., Handa, H. & Hazama, F. Experimentally induced cerebral aneurysms in rats. *Surg. Neurol.* **10**, 3–8 (1978). 684603

[5] Jamous, M. A. et al. Vascular corrosion casts mirroring early morphological changes that lead to the formation of saccular cerebral aneurysm: An experimental study in rats. *J. Neurosurg.* **102**, 532–535 (2005). 15796390 10.3171/jns.2005.102.3.0532

[6] Cayron, A. F. et al. Time-of-flight and black-blood MRI to study intracranial arteries in rats. *Eur. Radiol. Exp.* **8**, 3 (2024). 38191711 10.1186/s41747-023-00407-z PMC10774247

[7] Cayron, A. F. et al. Imaging of intracranial aneurysms in animals: A systematic review of modalities. *Neurosurg. Rev.* **46**, 56 (2023). 36786880 10.1007/s10143-023-01953-1 PMC9928939

[8] Pastor, G. et al. A general protocol of ultra-high resolution MR angiography to image the cerebro-vasculature in 6 different rats strains at high field. *J. Neurosci. Methods*. **289**, 75–84 (2017). 28694213 10.1016/j.jneumeth.2017.07.003

[9] Meyers, P. M. et al. Reporting standards for endovascular repair of saccular intracranial cerebral aneurysms. *J. Vasc Interv Radiol.* **20**, S435–S450 (2009). 19560031 10.1016/j.jvir.2009.03.004

[10] Makino, H. et al. Successful serial imaging of the mouse cerebral arteries using conventional 3-T magnetic resonance imaging. *J. Cereb. Blood Flow. Metab.* **35**, 1523–1527 (2015). 25920958 10.1038/jcbfm.2015.78 PMC4640342

[11] Lebas, H. et al. Imaging cerebral arteries tortuosity and velocities by transcranial doppler ultrasound is a reliable assessment of brain aneurysm in mouse models. *Stroke Vasc Interv Neurol.* **3**, e000476 (2023). 37496732 10.1161/SVIN.122.000476 PMC10368188

[12] Lee, R. M. Morphology of cerebral arteries. *Pharmacol. Ther.* **66**, 149–173 (1995). 7630927 10.1016/0163-7258(94)00071-a

[13] Kondo, S. et al. Cerebral aneurysms arising at nonbranching sites: An experimental study. *Stroke* **28**, 398–404 (1997). 9040697 10.1161/01.str.28.2.398

[14] Tutino, V. M. et al. Assessment of vascular geometry for bilateral carotid artery ligation to induce early Basilar terminus aneurysmal remodeling in rats. *Curr. Neurovasc Res.* **13**, 82–92 (2016). 26503026 10.2174/1567202612666151027143149 PMC5388353

[15] Schwyzer, L. et al. Scanning electron microscopy analysis of incidence and growth pattern of experimentally induced intracranial aneurysms in rat model. *Brain Hemorrhages*. **2**, 1–5 (2021).

[16] Aoki, T. et al. Rat model of intracranial aneurysm: Variations, usefulness, and limitations of the Hashimoto model. *Acta Neurochir. Suppl.* **127**, 35–41 (2020). 31407060 10.1007/978-3-030-04615-6_6

[17] Ciurica, S. et al. Arterial tortuosity. *Hypertension* **73**, 951–960 (2019). 30852920 10.1161/HYPERTENSIONAHA.118.11647

[18] Komura, S. et al. Computational fluid dynamics analysis features in aneurysm development in rats. *Neurol. Med. Chir. (Tokyo)*. **63**, 250–257 (2023). 37081649 10.2176/jns-nmc.2023-0005 PMC10325776

[19] Fernandez-Rodicio, S. et al. Perfusion-weighted software written in python for DSC-MRI analysis. *Front. Neuroinform*. **17**, 1202156 (2023). 37593674 10.3389/fninf.2023.1202156 PMC10431979

[20] Takao, H. et al. Comparing accuracy of cerebral aneurysm size measurements from three routine investigations: Computed tomography, magnetic resonance imaging, and digital Subtraction angiography. *Neurol. Med. Chir. (Tokyo)*. **50**, 893–899 (2010). 21030800 10.2176/nmc.50.893

[21] Han, H. J. et al. Formation, growth, or rupture of de novo intracranial aneurysms: long-term follow-up study of subarachnoid hemorrhage survivors. *Neurosurgery* **89**, 1104–1111 (2021). 34634821 10.1093/neuros/nyab364

[22] Tada, Y. et al. Roles of estrogen in the formation of intracranial aneurysms in ovariectomized female mice. *Neurosurgery* **75**, 690–695 (2014). 25181430 10.1227/NEU.0000000000000528 PMC4399640

[23] Aoki, T. & Nishimura, M. The development and the use of experimental animal models to study the underlying mechanisms of CA formation. *J. Biomed. Biotechnol.* **2011**, 535921 (2011). 10.1155/2011/535921 PMC3018658 21253583

[24] Miyamoto, T. et al. Site-specific elevation of interleukin-1beta and matrix metalloproteinase-9 in the Willis circle by hemodynamic changes is associated with rupture in a novel rat cerebral aneurysm model. *J. Cereb. Blood Flow. Metab.* **37**, 2795–2805 (2017). 27798272 10.1177/0271678X16675369 PMC5536789

[25] McDowell, K. P. et al. VasoMetrics: Unbiased spatiotemporal analysis of microvascular diameter in multi-photon imaging applications. *Quant. Imaging Med. Surg.* **11**, 969–982 (2021). 33654670 10.21037/qims-20-920 PMC7829163

[26] Ikedo, T. et al. Sequential inward bending of arterial bifurcations is associated with intracranial aneurysm formation. *World Neurosurg.* **129**, e361–e366 (2019). 31176059 10.1016/j.wneu.2019.05.153

[27] Kim, H. J. et al. How cerebral vessel tortuosity affects development and recurrence of aneurysm: Outer curvature versus bifurcation type. *J. Stroke*. **23**, 213–222 (2021). 34102756 10.5853/jos.2020.04399 PMC8189854

[28] Cornillie, P. et al. Corrosion casting in anatomy: Visualizing the architecture of hollow structures and surface details. *Anat. Histol. Embryol.* **48**, 591–604 (2019). 31120632 10.1111/ahe.12450

[29] James, A. et al. Hypertension and other etiological risk factors associated with the sublingual varices: A systematic review and meta-analysis. *J. Oral Biol. Craniofac. Res.* **14**, 720–729 (2024). 40276289 10.1016/j.jobcr.2024.09.014 PMC12020997

[30] Kang, M. et al. MRI visualization of whole brain macro- and microvascular remodeling in a rat model of ischemic stroke: A pilot study. *Sci. Rep.* **10**, 4989 (2020). 32193454 10.1038/s41598-020-61656-1 PMC7081185

