# Supplementary material for: Longitudinal MR angiographic evaluation of circle of Willis morphologic remodeling and induced aneurysms in Hashimoto rat cerebral aneurysm model
Source: Sci Rep. 2026 Feb 3;16:7094. doi: 10.1038/s41598-026-37369-2 (PMC12920620; doi:10.1038/s41598-026-37369-2)
Supplement: Supplementary file 1 — Supplementary Material 1 [file 41598_2026_37369_MOESM1_ESM.docx]

*Supplementary*

Supplementary Figure 1. Longitudinal changes in DBP (left) and MBP (right) in the induction and control groups. Red lines indicate the induction group (n=13), and blue lines indicate the control group (n=6). Following hypertension induction, both DBP and MBP were significantly elevated in the surgery group compared to the control group at all follow-up time points (W1, W4, and W12). Although a slight decrease in blood pressure was observed at W12, the hypertensive state was sustained over time, confirming the long-term hemodynamic stress induced by the surgical procedure. To evaluate whether the mean blood pressure at each time point significantly differed from the baseline (W0), one-sample t-tests were performed. Simulated normally distributed data centered around each time point mean were used for comparison against the W0 mean. Error bars represent standard deviation (SD). Significance was defined as p < 0.05.


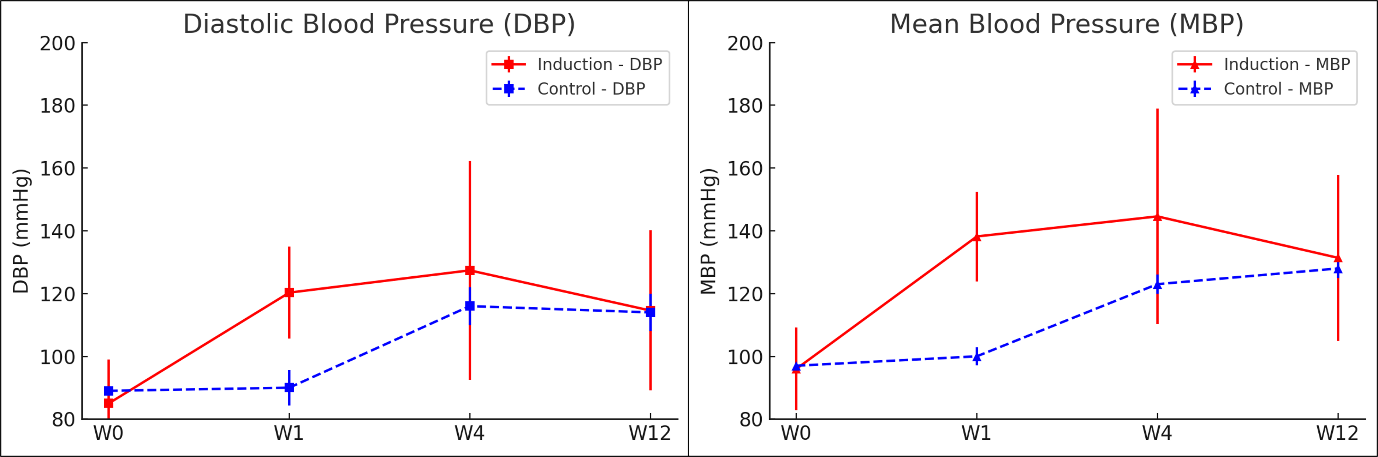


Supplementary Figure 2. Longitudinal BP trends for SBP, DBP, and MBP in the induction (n=13) and control groups (n=6). Red solid lines represent the induction group and blue dashed lines represent the control group. Each pressure type—SBP, DBP, and MBP—is plotted across four time points: W0 (baseline), W1, W4, and W12. Following unilateral carotid ligation and hypertension induction, all three blood pressure parameters showed significant elevation in the surgery group compared to the control group, particularly from W1 onward. The hypertensive effect was sustained over the follow-up period, despite a mild reduction at W12. Group comparisons were performed using one-sample t-tests. Error bars represent standard deviation (SD). Significance was defined as p < 0.05.


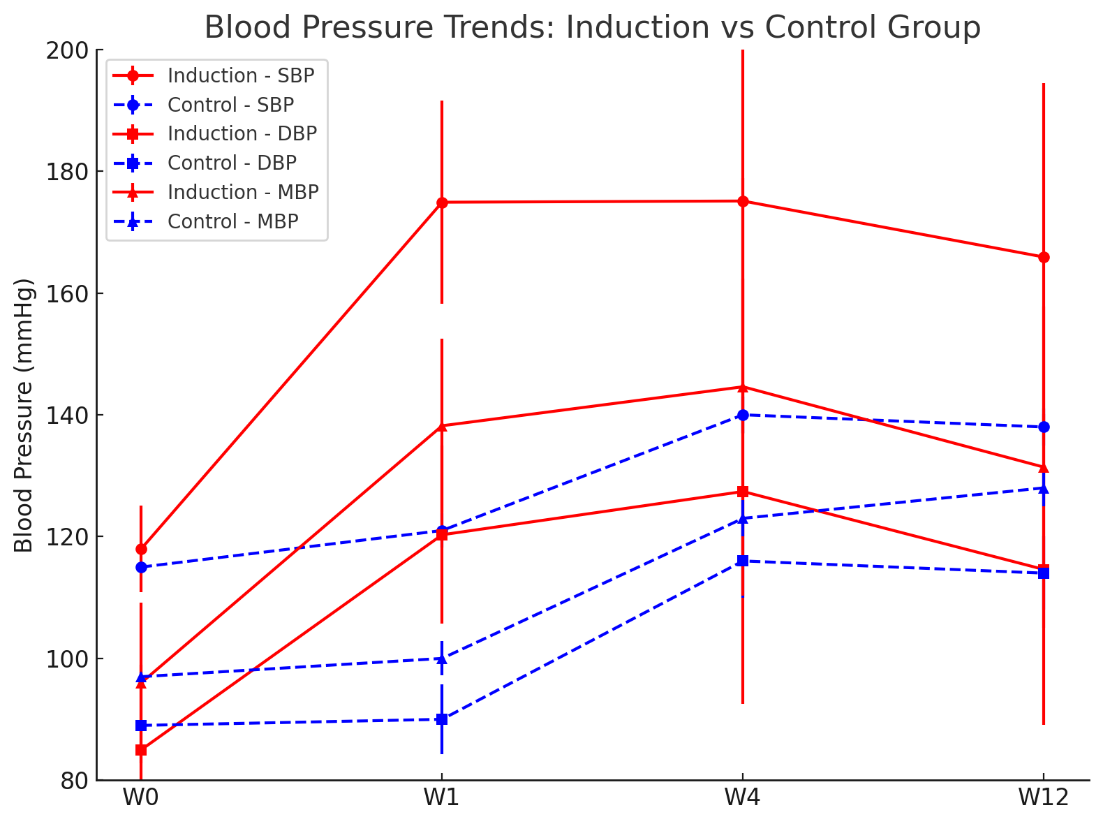


Supplementary Table 1. Baseline comparison of vascular diameters between left and right sides in the induction group (n=13) at W0, prior to carotid ligation surgery and hypertension induction. No significant side-to-side differences were observed across all analyzed vessel segments (P > 0.05), consistent with findings in the control group (n=6). These results indicate that vascular asymmetry in the induction group emerged only after unilateral carotid ligation and hypertension induction. Values represent the mean across animals for each segment.

To assess side-to-side differences in vascular diameter, linear mixed-effects models were fitted with time, side (left vs right), and their interaction (time × side) as fixed effects, and random intercepts for each subject to account for repeated measures. Post-hoc pairwise comparisons of estimated marginal means (LS-means) between left and right sides at each time point were performed with Tukey’s method for multiple comparisons adjustment. Statistical significance was set at a two-tailed alpha level of 0.05.

| **Vessel Segment** | **Time** | **L** | **R** | **P-value** |
| --- | --- | --- | --- | --- |
| **ACA A1** | W0 | 0.36 (0.32–0.40) | 0.38 (0.34–0.43) | 0.32 |
| **ACA A2** | W0 | 0.33 (0.28–0.38) | 0.30 (0.26–0.35) | 0.38 |
| **ICA** | W0 | 0.44 (0.40–0.48) | 0.46 (0.42–0.50) | 0.44 |
| **PCoA** | W0 | 0.37 (0.32–0.41) | 0.40 (0.35–0.44) | 0.27 |
| **PCA P1** | W0 | 0.22 (0.16–0.29) | 0.23 (0.16–0.30) | 0.87 |

Supplementary Table 2. Mean vessel diameters (in mm) of major COW segments in the induction group (n=13) measured at four time points: W0, W1, W4, and W12. Values represent the mean ± standard deviation (SD) across animals for each segment. The suffix “_R” indicates right-sided vessels, and “_L” indicates left-sided vessels. Notable asymmetric vascular remodeling was observed in the right ICA, left PCA P1, and left PCoA from W1 onward. A linear mixed-effects model was used for statistical analysis with group, time, and group-by-time interaction as fixed effects and random intercepts for animals. All statistical tests were performed using a two-tailed alpha level of 0.05.

| MEAN | A1_R | A1_L | A2_R | A2_L | ICA_R | ICA_L | BA | PCoA_R | PCoA_L | PCA_R | PCA_L | TI_R | TI_L |
| --- | --- | --- | --- | --- | --- | --- | --- | --- | --- | --- | --- | --- | --- |
| W0 | 0.39 | 0.38 | 0.30 | 0.34 | 0.47 | 0.45 | 0.50 | 0.40 | 0.37 | 0.23 | 0.22 | 1.55 | 1.57 |
| W1 | 0.47 | 0.44 | 0.32 | 0.34 | 0.57 | 0.47 | 0.59 | 0.43 | 0.48 | 0.22 | 0.38 | 1.64 | 1.75 |
| W4 | 0.46 | 0.43 | 0.34 | 0.35 | 0.58 | 0.48 | 0.56 | 0.44 | 0.47 | 0.26 | 0.50 | 1.71 | 1.89 |
| W12 | 0.49 | 0.37 | 0.33 | 0.28 | 0.58 | 0.41 | 0.55 | 0.39 | 0.41 | 0.21 | 0.40 | 1.96 | 2.12 |
